# To Restore the Cutting or Grinding Surfaces of Abraded Teeth with Gold

**Published:** 1902-07

**Authors:** F. Milton Smith

**Affiliations:** New York


### TO RESTORE THE CUTTING OR GRINDING SURFACES OF ABRADED TEETH WITH GOLD.[1]

BY DR. F. MILTON SMITH, NEW YORK.

FIRST separate the tooth to be restored from those on either side of it, so that a separation equal to Brown and Sharpe's gauge No. 25 is secured. If only one tooth is to be restored, grind off sufficiently from the cutting edge to accommodate whatever thickness of restoration you desire to make. The tip or restoration should be at least two thicknesses of No. 28 Brown and Sharp's gauge. After grinding down, as mentioned above, bevel the cutting edge very slightly all around, just sufficient to take off the sharp edge.

Drill holes into the cutting edge on either side of the pulp an eighth of an inch deep, more or less, as the case will permit. Usu-

---

[1] Read before The New York Institute of Stomatology, March 4, 1902.

ally cases requiring this work are not very sensitive, owing to recession of the pulp. These holes, for an upper central incisor, should be the diameter of the pin of an ordinary rubber tooth (not the head of it); smaller teeth, pins in proportion.

Take 34 to 36 Brown and Sharpe's gauge, pure gold, and burnish over the surface to be restored, being sure to have it run over the edge slightly, like the cover of a box. This is very important, and for it the separation was made. Ordinarily, a wood point is the best thing with which to adapt the gold to the tooth, the gold being very soft. A wad of bibulous paper pressed down with the wood is of assistance in the beginning. Then press with the wood point all over until a perfect adaptation is secured. When this has been done the dents in the gold show where the holes have been drilled in the tooth. Take a sharp point and pierce the gold for both pins. Take pure platinum wire exactly the size of the drill used. Make the ends small or slightly pointed, and press through the pure gold to the bottom of the hole. Often it is possible, without waxing, to withdraw the pin and gold together. If this can be done, hold under side of the pin with tweezers, apply minutest piece of 22-carat solder to the pin where it passes through the gold, hold over a small Bunsen flame or alcohol lamp and melt the solder. If the hole is no larger than the pin, the solder will not run to the under side.

Now place on tooth and carefully press to place as before with wood point, being very careful that the edges fit perfectly. Put second pin in place and dry gold and pin; then apply brittle wax (made by heating equal parts of beeswax and gum dammar and stirring the same together while hot, then allowing to cool), remove gold carefully, invest, and solder second pin. Replace on tooth, again carefully press to place all over, cut pins off just at height finished piece should be, apply brittle wax as high as pins, carefully remove, paint under side with rouge, and invest.

Have some 22-carat solder rolled very thin, cut size of tip and punch holes for pins. Put a minute quantity of borax on pure gold, drop solder over pins, prepare 22-carat plate same as piece of solder, drop in place, blow broad flame over the piece, and the solder will flow and the 22-carat gold will settle in its bed. Repeat the layers of solder and gold until you have the required height.

Remove investment, and if there is the remotest particle of solder on the under side, throw the piece into your scrap tray and begin again. If the minutest attention has been given to all the details, it will not be necessary to throw the piece away. Supposing it fits perfectly, take off and finish roughly out of the mouth. It is more pleasant for the patient. Apply dam, mix a small quantity of oxyphosphate the consistency of cream, carry to bottom of holes with a fine smooth broach wound with a particle of cotton, put drop of cement on end of tooth, press tip to its place, and let patient bite it solidly home. Keep it there until the cement is set. Finish at next sitting as perfectly as possible, and you have the strongest and best repair that can be made for the case.

With a little practice the same method can be successfully employed in incisor teeth, when both corners are broken off, necessitating the carrying of a gold filling if one is made from the gum line on the mesial down to and across the cutting edge and up to the gum line on the disto-approximal surface. If the pulp is living in such a case the piece will prove a good one. If dead, with so much of the tooth gone, probably a well-made porcelain crown would be better.

The success of this work depends upon absolute attention to details and perfect fit.

